# Retinoic Acid-Activated Ndrg1a Represses Wnt/β-catenin Signaling to Allow *Xenopus* Pancreas, Oesophagus, Stomach, and Duodenum Specification

**DOI:** 10.1371/journal.pone.0065058

**Published:** 2013-05-31

**Authors:** Tiejun Zhang, Xiaogang Guo, Yonglong Chen

**Affiliations:** 1 Key Laboratory of Regenerative Biology, Guangzhou Institutes of Biomedicine and Health, Chinese Academy of Sciences, Guangzhou, China; 2 Guangdong Provincial Key Laboratory of Stem Cell and Regenerative Medicine, Guangzhou, China; 3 Graduate University of Chinese Academy of Sciences, Beijing, China; Laboratoire de Biologie du Développement de Villefranche-sur-Mer, France

## Abstract

How cells integrate multiple patterning signals to achieve early endoderm regionalization remains largely unknown. Between gastrulation and neurulation, retinoic acid (RA) signaling is required, while Wnt/β-catenin signaling has to be repressed for the specification of the pancreas, oesophagus, stomach, and duodenum primordia in *Xenopus* embryos. In attempt to screen for RA regulated genes in *Xenopus* endoderm, we identified a direct RA target gene, N-myc downstream regulated gene 1a (*ndrg1a*) that showed expression early in the archenteron roof endoderm and late in the developing pancreas, oesophagus, stomach, and duodenum. Both antisense morpholino oligonucleotide mediated knockdown of *ndrg1a* in *Xenopus laevis* and the transcription activator-like effector nucleases (TALEN) mediated disruption of *ndrg1* in *Xenopus tropicalis* demonstrate that like RA signaling, Ndrg1a is specifically required for the specification of *Xenopus* pancreas, oesophagus, stomach, and duodenum primordia. Immunofluorescence data suggest that RA-activated Ndrg1a suppresses Wnt/β-catenin signaling in *Xenopus* archenteron roof endoderm cells. Blocking Wnt/β-catenin signaling rescued Ndrg1a knockdown phenotype. Furthermore, overexpression of the putative Wnt/β-catenin target gene Atf3 phenocopied knockdown of Ndrg1a or inhibition of RA signaling, while Atf3 knockdown can rescue Ndrg1a knockdown phenotype. Lastly, the pancreas/stomach/duodenum transcription factor Pdx1 was able to rescue Atf3 overexpression or Ndrg1a knockdown phenotype. Together, we conclude that RA activated Ndrg1a represses Wnt/β-catenin signaling to allow the specification of pancreas, oesophagus, stomach, and duodenum progenitor cells in *Xenopus* embryos.

## Introduction

The regionalization of endoderm occurs concurrently with its formation during gastrulation. By the end of gastrulation, broad antero-posterior (AP) domains within the endoderm have been established, as reflected by the anterior expression of *Hhex*, *vpp1*, *Sox2*, and *Foxa2* and the posterior expression of the *caudal type homeobox* genes *Cdx1*, *2*, and *4* [1], [2], [3]. Eventually, the endoderm gives rise to the epithelia of the respiratory and gastrointestinal tracts and their associated organs, such as the thyroid, lungs, pancreas, liver and gall bladder. The lineage tracing for vertebrate endodermal organogenesis is largely dependent on the vital dye labeling-based fate mapping studies [4], [5], [6], [7], [8]. The early expression of *hhex* and *vpp1* in *Xenopus* embryos might serve to reflect the precursors for the liver and ventral pancreas, respectively [3]. So far, there are few specific marker genes reported, which can demarcate precursors for dorsal pancreas, oesophagus, stomach, and duodenum during gastrulation and neurulation.

Retinoic acid (RA) signaling plays a conserved role in the AP patterning of vertebrate endoderm as early as gastrulation [9]. Studies in frog, avian, and mice indicate that the RA signaling is specifically required for the formation of dorsal pancreas, oesophagus, stomach, and duodenum primordia [10], [11], [12], [13], [14], [15]. In zebrafish, RA signaling before the end of gastrulation is required for the initial development of both hepatic and pancreatic endoderm [16]. RA activated expression of RA-degrading enzyme *cyp26a1* in the anterior trunk endoderm in turn modulates RA signaling and defines the anterior boundary of pancreatic field in zebrafish embryos [17]. *mnx1* is identified as an RA downstream gene that promotes beta cell formation in the developing zebrafish endocrine pancreas [18], while RA downstream gene *exdpf* regulates fish exocrine pancreas development [19]. The endodermal RA target genes that mediate the early activities of RA signaling to specify the pancreas, oesophagus, stomach, and duodenum anlagen proper remain to be identified.

Canonical Wnt signaling also plays an important role in the AP patterning of vertebrate endoderm. In mice, compound knockout of *Tcf1* and *Tcf4* led to an anterior transformation of duodenum into stomach with little or no intestine developed [20]. The stomach mesenchymal transcription factor Barx1-mediated secretion of the Wnt antagonists SFRP1 and SFRP2 is required to inhibit endodermal Wnt/β-catenin signaling and thus to permit specification of the stomach epithelium [21]. Consistently, in *Xenopus*, Wnt/β-catenin signaling must be repressed in anterior endoderm between gastrula and early somite stages of development to allow the formation of the liver as well as the pancreas, stomach, and duodenum primordia [22], [23]. The secreted Wnt antagonist *sfrp5* expressed in the ventral foregut endoderm coordinates the liver and ventral pancreas specification by antagonizing both canonical and noncanonical Wnt signaling [24], [25]. In addition, it has been shown that RA can repress *Xenopus* blastula Wnt/β-catenin signaling [26].

N-myc downstream regulated gene 1 (NDRG1) belongs to the NDRG protein family consisting of four members, NDRG1–4, which are characterized by containing a NDR domain and an α/β hydrolase-fold motif [27]. *NDRG1* is an RA-inducible gene in various cell lines and human patients [28], [29], [30], [31]. *Ndrg1* deficiency in mice leads to Schwann cell dysfunction, suggesting that NDRG1 is essential for maintenance of the myelin sheaths in peripheral nerves [32], [33]. *Ndrg1*-null mice also showed impaired mast cell maturation and degranulation and attenuated allergic responses [34]. In *Xenopus*, *ndrg1* was identified as a gene enriched for expression in endoderm and pronephros [35], [36]. A recent study using cancer cell lines reveals that the tumor metastasis suppressor gene, NDRG1, represses Wnt/β-catenin signaling by directly interacting with the Wnt receptor, LRP6, consequently causing the suppression of Wnt/β-catenin target gene, *ATF3* expression [37]. Activating transcription factor 3 (ATF3) belongs to the ATF/cyclic AMP responsive element binding family of basic leucine zipper transcription factors. It is an adaptive response gene and can act both as a transcriptional activator or repressor depending on the cell type and stimulus [38], [39], [40]. *ATF3* knockout mice developed normally and did not show any discernable phenotypes under normal conditions [41]. In contrast, transgenic mice expressing ATF3 in the liver, pancreatic ductal epithelium, or pancreatic β cells led to liver dysfunction, defects in endocrine pancreas development, or islet dysfunction, respectively [41], [42], [43].

In this study, we used DNA microarray in combination with whole mount in situ hybridization to screen RA regulated genes in *Xenopus* endoderm. *ndrg1a* was identified and verified being directly activated by RA in the archenteron roof endoderm and late in the developing pancreas, oesophagus, stomach, and duodenum. We provide evidences implicating that RA-activated Ndrg1a represses Wnt/β-catenin signaling in the archenteron roof endoderm cells and consequently releases the inhibitory effect of Wnt/β-catenin signaling activities on the formation of pancreas, oesophagus, stomach, and duodenum.

## Results

### *ndrg1a*, an RA Regulated Gene, is Expressed Early in the Endodermal Archenteron Roof

**Figure 1:**
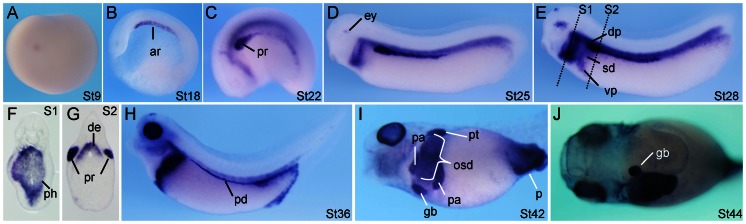
*Xenopus ndrg1a* is expressed early in the archenteron roof endoderm and late in pancreas, oesophagus, stomach, and duodenum primordia, as revealed by whole mount in situ hybridization. (A) Lateral view of a blastula. (B) Whole mount in situ hybridization on bisected stage 18 neurula, anterior toward the left. (C–E) Lateral view, head toward the left. Note that the foregut *ndrg1a* signal in E covers pharynx, oesophagus, pancreas, stomach, and duodenum. (F, G) Transversal sections of a stage 28 embryo at the levels illustrated by the dashed lines in E. (H, I) Lateral view, head toward the left. (J) Ventral view, head toward the left. Abbreviations: ar, archenteron roof endoderm; de, dorsal endoderm; dp, dorsal pancreatic bud; ey, eye; gb, gall bladder; osd, oesophagus, stomach and duodenum anlagen; p, proctodaeum; pa, pancreas; pd, pronephric duct; ph, pharynx; pr, pronephric anlage; pt, pronephric proximal tubules; vp, ventral pancreatic bud.

With respect to endodermal organogenesis, *Xenopus* embryos treated with an RA antagonist, BMS453, before the end of gastrulation displayed specific loss of the dorsal pancreas and part of ventral pancreas, stomach, and duodenum [10]. To identify RA regulated genes in endoderm, we first did microarray analysis comparing gene expression levels between wild-type and RA-deficient embryos, which were treated with BMS453 at the beginning of gastrulation and were collected at stages 12, 23, and 34, respectively. The data obtained indicate that genes down-regulated more than 2-fold at three different stages showed limited overlap (Fig. S1 and Table S1). In embryos collected at stage 23, there are 138 genes that were down-regulated more than 2-fold upon BMS453 treatment. According to the 138 genes’ structural and functional information available in the NCBI database, we gave priorities to transcription factors, kinases, RNA binding proteins, transmembrane proteins, as well as genes showing endodermal expression, and thus chose 9 genes (Table S1) to further analyze their embryonic expression patterns by whole mount in situ hybridization. One of them, *ndrg1a*, showed specific expression early in the archenteron roof endoderm (Fig. 1B), which was not detected by previous studies, presumably due to insufficient sensitivity of the whole mount in situ hybridization technique in previous publications [35], [36]. In agreement with the report from the Zorn laboratory [35], our data indicate that *ndrg1a* transcripts were detected in dorsal endoderm at early tail bud stage of development (Fig. 1C, D, E, G). At late tail bud and tadpole stages of development, in addition to its expression in eye, proctodaeum precursors, and pronephros, *ndrg1a* showed specific expression in the pharynx, oesophagus, pancreas, stomach, and duodenum (Fig. 1E, F, H, I, J). We were unable to detect *ndrg1a* expression in the liver [35]. Instead, a clear *ndrg1a* signal was observed in developing gall bladder (Fig. 1I, J).

### *ndrg1a* Expression in Archenteron Roof Endoderm Cells is Directly Activated by RA

**Figure 2:**
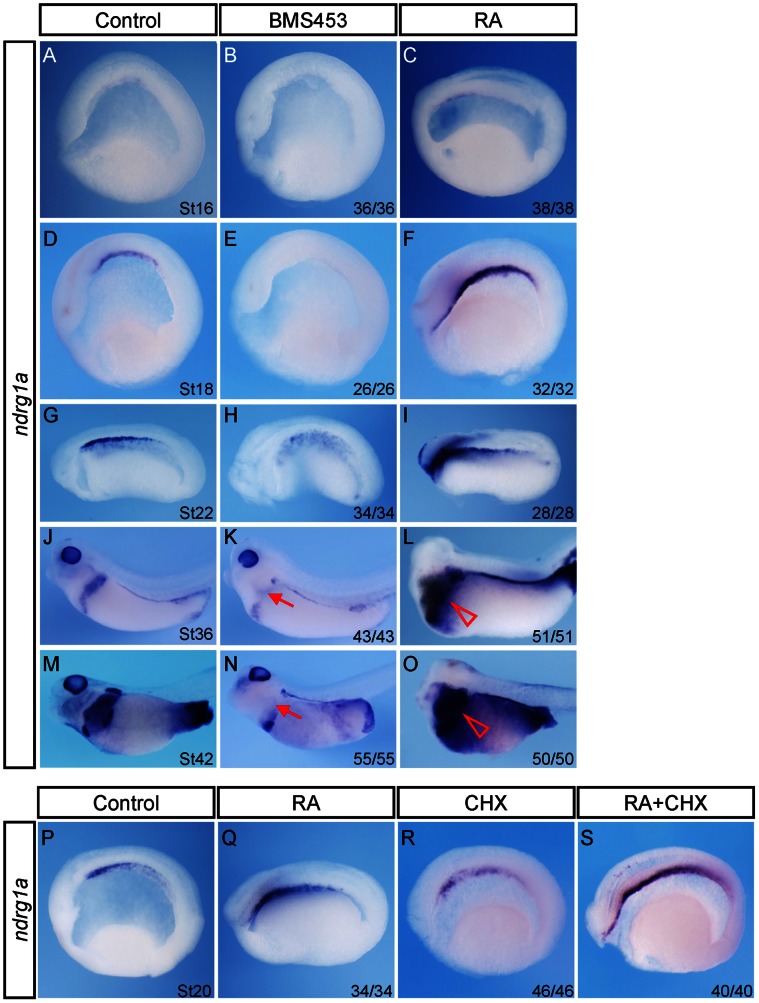
RA directly activates *ndrg1a* expression in *Xenopus* archenteron roof endoderm cells. (A–O) RA signaling is necessary and sufficient to activate *ndrg1a* expression in the archenteron roof endoderm cells as well as in the pancreas, oesophagus, stomach, and duodenum primordia. Embryos were treated with 0.25 µM BMS453 or 5 µM RA at stage 10 for one hour, collected at stages indicated in the control panels, and subjected to whole mount in situ hybridization analyses of *ndrg1a* expression. (A–I) Whole mount in situ hybridization on bisected embryos, anterior toward the left. (J–O) Lateral view, head toward the left. Red arrows in panels K and N and red triangles in panels L and O point to the loss or expansion of *ndrg1a* expression in pancreas, oesophagus, stomach, and duodenum upon BMS453 or RA treatment, respectively. (P–S) RA directly activates *ndrg1a* expression in *Xenopus* archenteron roof endoderm cells. Embryos were treated with cycloheximide (CHX, 10 µg/ml), 5µM RA, or both at stage 15 for one hour, fixed at stage 20, and bisected for whole mount in situ hybridization analyses of *ndrg1a* expression. For the combined treatment, CHX was added 15 min before the application of RA. Anterior is toward the left. The numbers of embryos showing the illustrated phenotypes are given in the corresponding images.

To further verify the microarray data, we analyzed *ndrg1a* expression in BMS453 and RA treated embryos by whole mount in situ hybridization. Upon BMS453 treatment, no *ndrg1a* transcripts were detected in archenteron roof endoderm cells (Fig. 2E) and only traces of *ndrg1a* mRNA remained in dorsal endoderm of stage 22 embryos (Fig. 2H). Later on, *ndrg1a* expression was completely repressed in dorsal pancreas, oesophagus, stomach, and duodenum and was partially inhibited in ventral pancreas (Fig. 2K, N). In contrast, another RA antagonist BMS493 can only partially inhibit *ndrg1a* expression in pancreas, oesophagus, stomach, and duodenum [11]. In addition, *ndrg1a* expression in pronephric proximal tubules was also severely inhibited by BMS453 (Fig. 2N). It should be noted that *ndrg1a* expression in eye, brain, and gall bladder was less affected upon BMS453 treatment (Fig. 2K, N). It is a common phenomenon that for genes expressing in pancreas as well as in eye and central nervous system, such as *ndrg1a*, *esr10*, *neurod*, and *ptf1a/p48*, only their pancreatic expression is inhibited upon BMS453 treatment (Fig. 2K, N and [10]). Conversely, application of RA to *Xenopus* embryos induced significant expansion of *ndrg1a* expression domains in archenteron roof endoderm (Fig. 2F), dorsal endoderm (Fig. 2I), pancreas, oesophagus, stomach, and duodenum (Fig. 2L, O). Thus, RA signaling is necessary and sufficient to activate *ndrg1a* expression in the archenteron roof endoderm cells.

To address if RA can directly regulate *ndrg1a* expression, we treated *Xenopus* embryos with cycloheximide (CHX), a protein synthesis inhibitor, 15 minutes before the application of RA. Our data indicate that RA induced expansion of *ndrg1a* expression in archenteron roof endoderm was not affected by CHX (Fig. 2S), supporting the notion that RA directly activates *ndrg1a* expression in archenteron roof endoderm cells. Indeed, an RA response element was identified in *Xenopus tropicalis ndrg1* promoter region (AGTTCAacAGTTCA −1496bp to −1509bp). Unlike *Xenopus laevis*, the diploid *Xenopus tropicalis* has one *ndrg1* gene.

### *ndrg1a* Knockdown in *Xenopus* Embryos Phenocopies BMS453 Treatment

**Figure 3:**
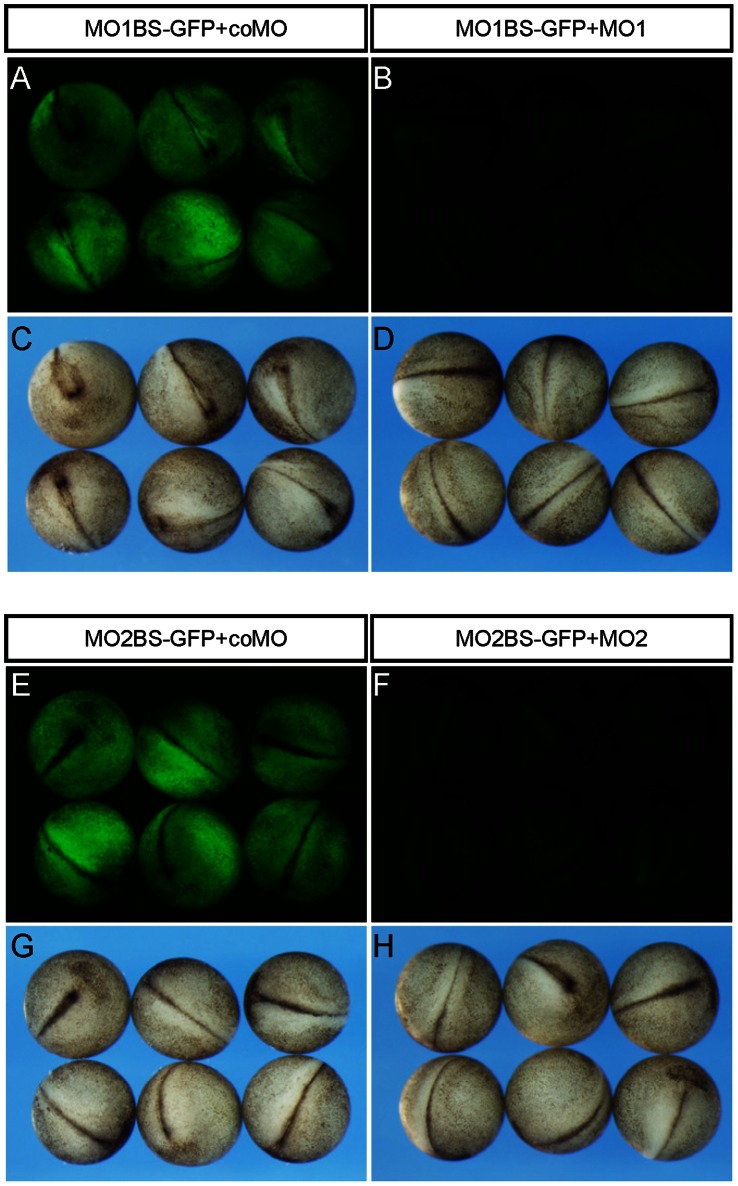
The efficiency of *ndgr1a* MO1 and MO2 was verified in vivo. (A–D) Fertilized *Xenopus* eggs were co-injected with 3.5 picomoles of coMO or *ndrg1a* MO1 in combination with an mRNA containing MO1 binding site followed by GFP coding sequence (MO1BS-GFP) and evaluated for live GFP translation at stage 18 with an Olympus SZX16 fluorescence microscope. (A, B) GFP. (C, D) Bright field view. (E–H) *ndrg1a* MO2 specifically inhibited the translation of GFP following its binding site (MO2BS-GFP), as revealed by the in vivo assay as above. (E, F) GFP. (G, H) Bright field view.

To generate *ndrg1a* morphants, we purchased two *ndrg1a* antisense morpholino oligos (MO) from Gene Tools. One covers the ATG start codon (MO1) and the other (MO2) locates in the 5′ untranslated region of the *ndrg1a* cDNA. Their efficiency was tested by an in vivo assay, in which GFP coding sequence was fused to a MO’s binding site and the resultant mRNA was co-injected with the MO into *Xenopus* fertilized eggs to evaluate live GFP translation in embryos. Both MO1 (Fig. 3B) and MO2 (Fig. 3F) were able to efficiently inhibit the translation of GFP open reading frame following the corresponding MO1 or MO2 binding sequences, respectively, while control MO (coMO) failed to do so (Fig. 3A, E).

To test if Ndrg1a can functionally mediate RA signaling to specify foregut endoderm, we injected MO1, MO2, or coMO separately into the vegetal part of all four blastomeres of 4-cell stage embryos, collected the embryos at stages 36 and 42, and analyzed with a panel of endodermal marker genes. *ptf1a*/*p48* is specific for pancreatic precursor cells during early embryogenesis and later becomes restricted to the exocrine pancreas [10], [44], [45]. *pdx1* specifically demarcates developing pancreas, stomach, and duodenum [46]. *insulin*, an endocrine marker gene, is exclusively expressed in the dorsal pancreatic bud of *Xenopus* tail bud stage embryos [47], [48]. *pdip* is an exocrine pancreas specific marker gene [49]. *sox2* marks the oesophagus, stomach and an anterior portion of the duodenum [50]. *darmin* is an intestine specific marker gene [35], [51]. *hhex* serves as a liver and thyroid marker gene [52]. *nkx2*.*1* marks developing lung and thyroid [53].

**Figure 4:**
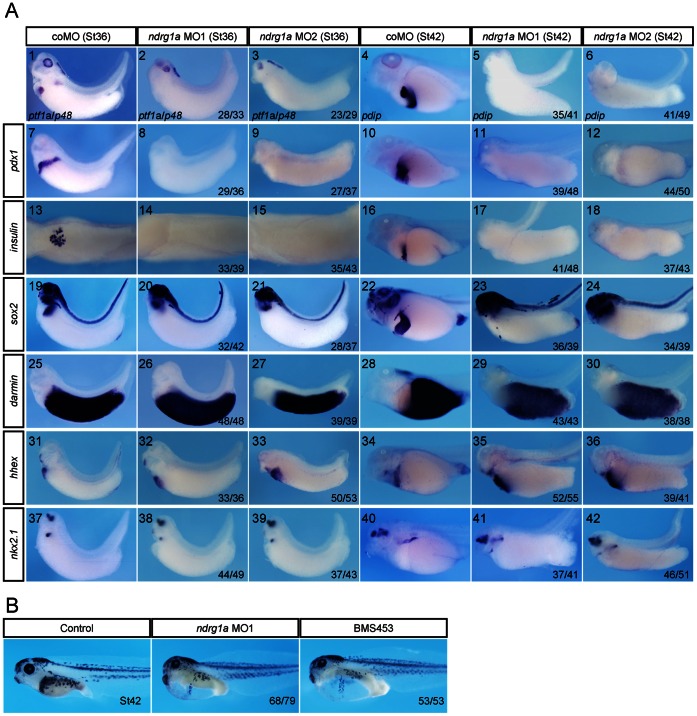
*ndrg1a* knockdown in *Xenopus* embryos specifically disturbed pancreas, oesophagus, stomach, and duodenum formation, which phenocopied BMS435 treatment. (A) 3.5 picomoles of coMO, *ndrg1a* MO1 or *ndrg1a* MO2 was vegetally injected into all four blastomeres at four cell stage of development and collected at stages 36 and 42 for whole mount staining with marker genes indicated on the left side or in the images 1–6. (A1–12 and A16–42) Lateral view, head toward the left. (A13–15) Dorsal view. The dorsal structures, such as the neural tube, notochord, and somites were removed after whole mount in situ hybridization. (B) Morphology of *ndrg1a* MO1 injected and BMS453 treated embryos collected when control siblings developed to stage 42. The numbers of embryos showing the illustrated phenotypes are given in the corresponding images.

The data obtained indicate that MO1 and MO2 equally efficiently abolished *insulin*, *ptf1a*/*p48*, *pdip*, *pdx1*, and *sox2* expression in pancreas, oesophagus, stomach, and duodenum, but had minor influence on *darmin*, *hhex*, and *nkx2*.*1* expression in intestine, liver, thyroid, and lung, which is very similar to the effect of BMS453 (Fig. 4A and [10]). The only difference is that no ventral pancreatic expression of *ptf1a*/*p48*, *pdip*, or *pdx1* was observed in *ndrg1a* morphants, but traces of *ptf1a*/*p48*, *pdip*, and *pdx1* expression in the ventral pancreatic buds were maintained in BMS453 treated embryos (Fig. 4A and [10]). The morphological phenotype of *ndrg1a* knockdown also resembles that of BMS453 treatment. Embryos injected with MO1 or MO2 appeared normal before stage 40 and subsequently displayed severe gut malformations with a loss of gut coiling and formation of edema (Fig. 4B and [10]). As MO1 and MO2 caused identical phenotypes, we used MO1 to carry out the rest studies. Together, these data suggest that RA and Ndrg1a are in the same genetic hierarchy in controlling pancreas, oesophagus, stomach, and duodenum development.

**Figure 5:**
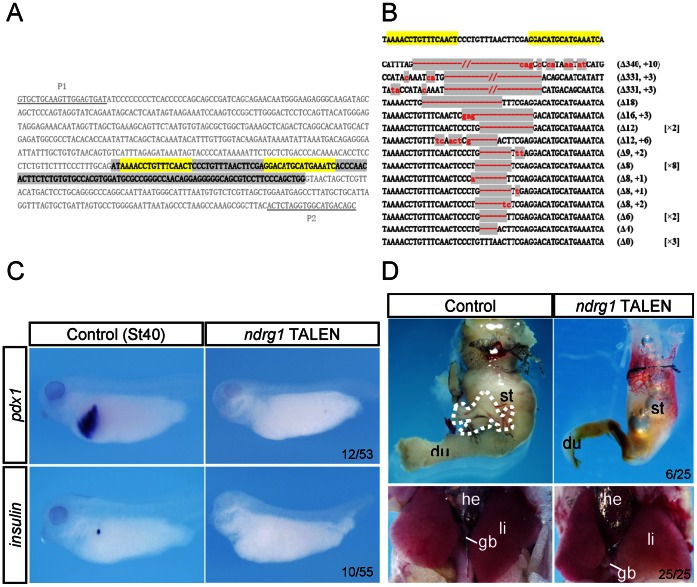
TALEN mediated disruption of *ndrg1* in *Xenopus tropicalis* confirmed MO mediated *ndrg1a* knockdown phenotype in *Xenopus laevis*. (A) The TALEN targeting site in *Xenopus tropicalis ndrg1* locus (GenBank accession no. NM_001008145.1) was designed in the third exon highlighted in gray with the flanking introns in plain text. The TALEN recognition sequences are highlighted in yellow. The underlined sequences are PCR primers (P1 and P2) used for the evaluation of the gene targeting efficiency. (B) Somatic mutations induced by *ndrg1* TALENs in *Xenopus tropicalis* embryos. Deletions (Δ) are indicated by red dashes and insertions (+) by lowercase red letters against a gray background. The numbers in parentheses show the number of deleted or inserted base pairs. The frequency of the mutation in the sequenced samples is shown in the square brackets. Up to 90% (28/31) of *ndrg1* loci sequenced were disrupted. (C) Whole mount in situ hybridization analysis of *pdx1* and *insulin* expression in stage 40 *Xenopus tropicalis* embryos with or without injection of *ndrg1* TALEN mRNAs. All images are lateral view with head toward the left. The numbers of embryos showing the illustrated phenotypes are given in the corresponding images. (D) *Xenopus tropicalis* froglets once subjected to the injection of *ndrg1* TALEN mRNAs showed pancreas, stomach, and duodenum hypoplasia (upper right image), while their liver and gall bladder developed normally (lower right image). Among 25 froglets sacrificed and dissected, 6 showed pancreas aplasia as illustrated in D (upper right image) and the rest showed severe pancreas hypoplasia, while all of them showed stomach and duodenum hypoplasia. The pancreas in control froglet is outlined by white dashed lines. Abbreviations: du, duodenum; gb, gall bladder; he, heart; li, liver; st, stomach.

Injection of even 4 ng of *ndrg1a* mRNA into *Xenopus* embryos caused neither morphological abnormality nor expression alterations of the marker genes analyzed (Fig. S2A, B). Co-injection of *ndrg1a* mRNA with its MOs could not rescue the MO phenotype either (data not shown). It seems that we were unable to get functional Ndrg1a protein in *Xenopus* embryos through the routine mRNA injection protocol that works for most if not all other genes analyzed. An Ndrg1a-GFP construct demonstrated that the protein was produced (Fig. S3).We have recently established TALEN mediated gene targeting in *Xenopus* [54]. To further verify the MO phenotype, we designed *ndrg1* TALENs within the third exon of *Xenopus tropicalis ndrg1* gene (Fig. 5A), which efficiently caused somatic mutations at the targeted loci (Fig. 5B). Consistent with the MO phenotype obtained in *Xenopus laevis* embryos, *insulin* and *pdx1* expression was severely inhibited in *ndrg1* TALEN mRNA injected *Xenopus tropicalis* embryos (Fig. 5C), albeit with a lower frequency in comparison to the MO injected ones, which happened also to the application of *ptf1a/p48* TALENs in our previous study [54]. A stable *ndrg1* knockout frog line is yet to be established. We dissected 25 froglets that showed pancreas, stomach, and duodenum hypoplasia or even aplasia but with normal liver and gall bladder developed (Fig. 5D). Thus, these data confirmed the *ndrg1a* MO phenotype obtained in *Xenopus laevis*.

### RA Activated Ndrg1a Represses Wnt/β-catenin Signaling in Archenteron Roof Endoderm Cells to Allow Pancreas, Oesophagus, Stomach, and Duodenum Specification

**Figure 6:**
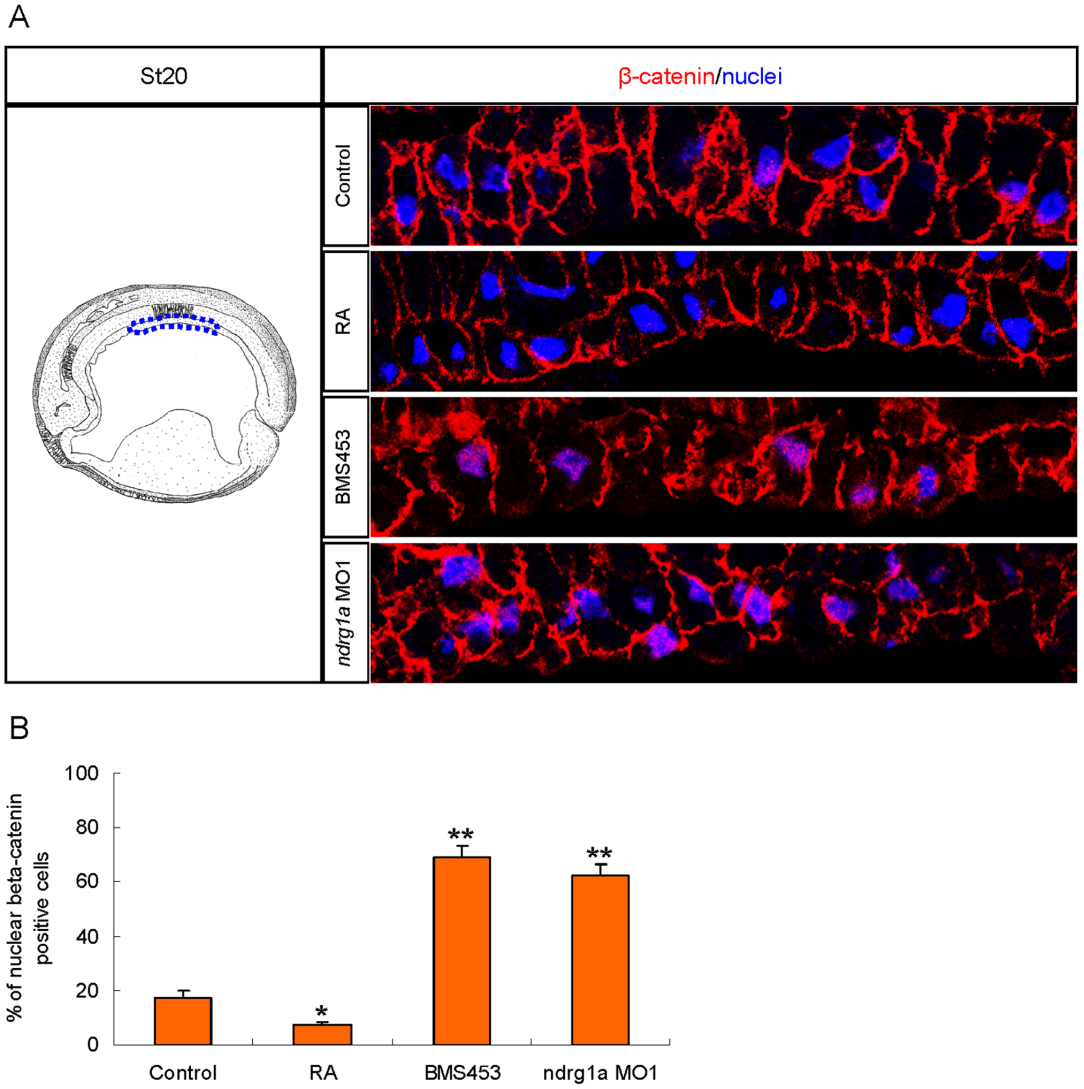
Nuclear β-catenin localization in archenteron roof endoderm cells appears to be suppressed by RA activated Ndrg1a in *Xenopus laevis*. Embryos were treated with 5 µM RA, 0.25 µM BMS453 for one hour at stage 10, or vegetally injected with 3.5 picomoles of *ndrg1a* MO1 at four cell stage and collected at stage 20 for immunofluorescence. (A) Left panel is a schematic drawing illustrating a midsagittal section of stage 20 embryos (after Hausen and Riebesell [71]). The dashed blue lines outline the archenteron roof endoderm where *ndrg1a* is expressed. Right panels are representative immunofluorescence images showing β-catenin signals (red channel) and DAPI staining (blue channel) in the outlined archenteron roof endoderm cells. (B) Quantification data obtained from three independent experiments. Nine embryos in total (three for every experiment) from each group were sectioned to evaluate the mean percentage of β-catenin positive cells in the outlined archenteron roof endoderm illustrated in the left panel of A. For each embryo, the outlined archenteron roof endoderm cells in the 30 consecutive parasagittal sections central to the median plane were scanned for nuclear β-catenin signals. *, *p*<0.05. **, *p*<0.01 (Student’s *t*-test, two-tailed distribution).

Mechanistically, NDRG1 can interact with Wnt receptor LRP6 and block Wnt/β-catenin signaling in cancer cell lines [37]. To ask if RA activated Ndrg1a can repress Wnt/β-catenin signaling in *Xenopus* embryos, we compared nuclear β-catenin levels in *ndrg1a* positive archenteron roof endoderm cells of stage 20 embryos that were subjected to RA, BMS453 treatment, or MO1 injection. Immunolocalization of nuclear β-catenin is a reliable method to characterize Wnt/β-catenin signaling activity in *Xenopus* embryos [55]. In the selected area of archenteron roof endoderm, a few cells showed nuclear β-catenin staining under normal condition, which became further less upon RA treatment. In contrast, the number of nuclear β-catenin positive cells in the archenteron roof endoderm significantly increased upon BMS453 treatment, or MO1 injection (Fig. 6A, B). Together, these data suggest that RA and Ndrg1a repress Wnt/β-catenin signaling in *Xenopus* archenteron roof endoderm cells.

**Figure 7:**
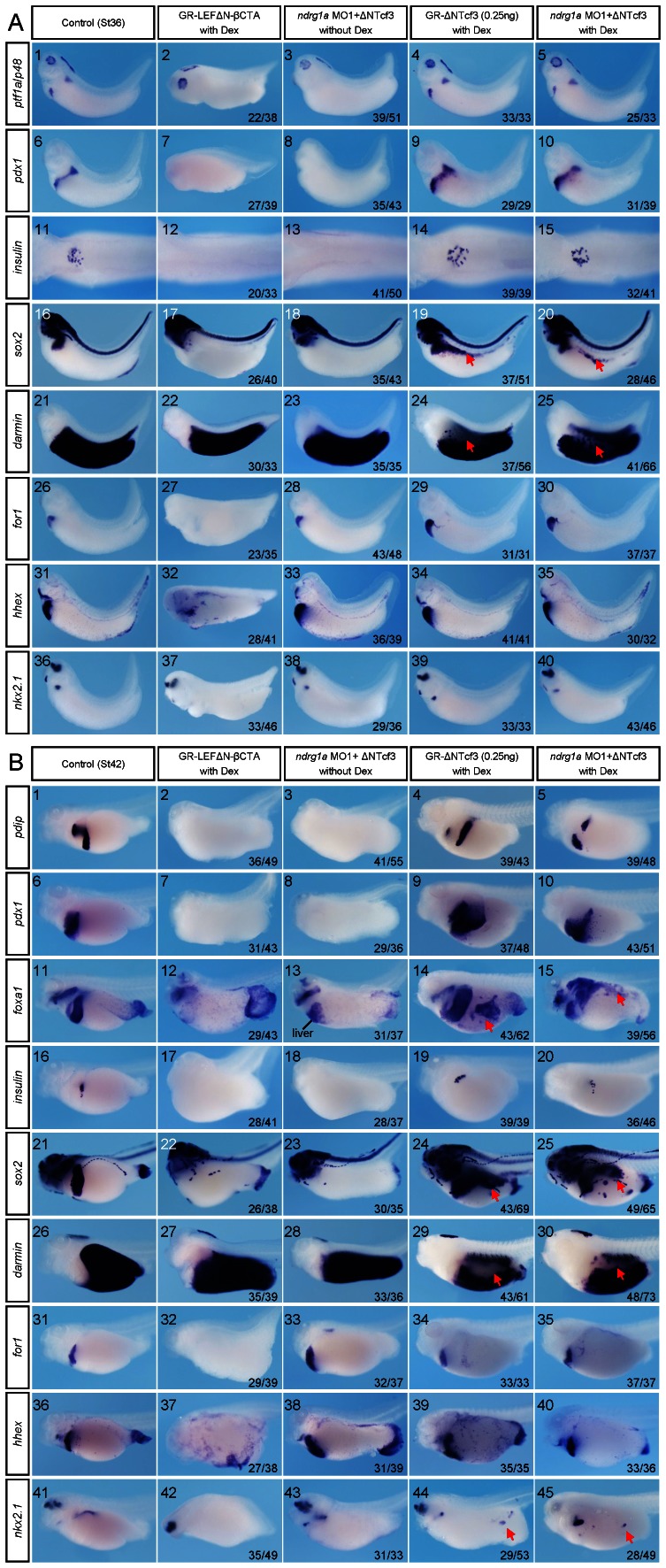
Ndrg1a represses Wnt/β-catenin signaling, thus specially allowing pancreas, oesophagus, stomach, and duodenum specification. (A, B) *Xenopus laevis* embryos were vegetally injected with the reagents indicated on the top, treated with 10 µM dexamethasone (Dex) at stage 15, collected at stages 36 (A) and 42 (B), and subjected to whole mount staining with probes indicated on the left side. Doses of the reagents injected are as follows: *ndrg1a* MO1, 3.5 picomoles; GR-ΔNTcf3 mRNA, 0.25 ng; GR-LEFΔN-βCTA mRNA, 0.5 ng. Red arrows in images A19, 20, 24, 25, B14, 15, 24, 25, 29, 30, 44, and 45 point to either ectopic or loss of expression of marker genes indicated upon inhibition of Wnt/β-catenin signaling. (A11–15) Dorsal view. The dorsal structures, such as the neural tube, notochord, and somites were removed after whole mount in situ hybridization. All the rest images in A and B are lateral view with head toward the left. The numbers of embryos showing the illustrated phenotypes are given in the corresponding images.

To test if RA, Ndrg1a, and Wnt/β-catenin signaling are functionally linked in the same cascade in controlling pancreas, oesophagus, stomach, and duodenum specification, we co-injected *ndrg1a* MO1 with GR-ΔNTcf3, a dominant negative form of Tcf3 fused with the hormone-binding domain of the glucocorticoid receptor serving to repress Wnt/β-catenin signaling upon dexamethasone induction [56]. Our data indicate that repressing Wnt/β-catenin signaling rescued *ndrg1a* knockdown phenotype (Fig. 7A, B). Ectopic activation of *nkx2*.*1* (Fig. 7B44, 45), *sox2* (Fig. 7A19, 20 and B24, 25), and *foxa1* (Fig. 7B14, 15), a forkhead transcription factor marking stomach, duodenum, and proctodaeum [44], is correlated with the loss of *darmin* expression (Fig. 7A24, 25, 7B29, 30) upon injection of 0.25 ng of GR-ΔNTcf3 mRNA. In line with the previous study [22], higher dose (0.8 ng) of GR-ΔNTcf3 mRNA is required to induce ectopic expression of *pdx1* and *for1*,a liver specific marker gene [22], [57], in *Xenopus* embryos (data not shown).

In full agreement with the earlier study [22], activation of canonical Wnt signaling in *Xenopus* endoderm by vegetal injection of 0.5 ng of GR-LEFΔN-βCTA mRNA containing Lef1 DNA-binding domain and the β-catenin transactivation domain [58] into 4 blastomeres of the 4-cell stage embryos resulted in identical phenotype caused by Ndrg1a knockdown in the context of pancreas, oesophagus, stomach, and duodenum development (Fig. 7A, B). The suppression effect of Wnt/β-catenin signaling on the formation of liver (Fig. 7A27, 32, B32, 37) and lung (Fig. 7A37, B42), was not seen in Ndrg1a morphants, suggesting that the early repression of Wnt/β-catenin signaling in the territory of liver and lung forming endoderm is executed by factors other than RA activated Ndrg1a. Taken together, these data suggest that Wnt/β-catenin signaling is downstream of Ndrg1a and is repressed by Ndrg1a to allow the specification of pancreas, oesophagus, stomach, and duodenum.

### Atf3, a Putative Wnt/β-catenin Target Gene in *Xenopus* Embryos, Specifically Inhibits *Xenopus* Pancreas, Oesophagus, Stomach, and Aduodenum Specification

**Figure 8:**
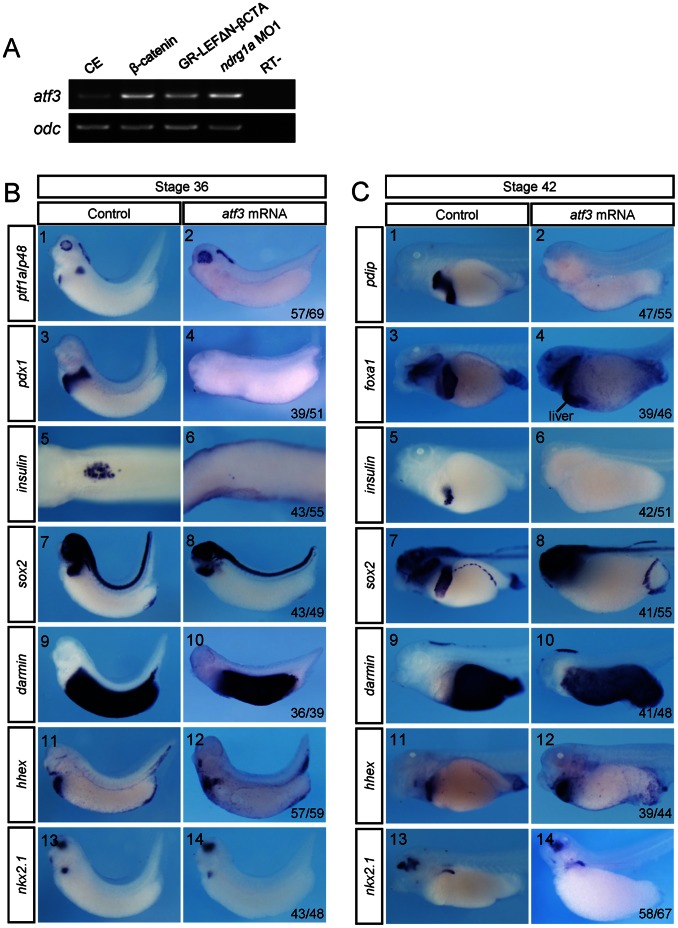
Overexpression of Atf3 phenocopied *ndrg1a* knockdown. (A) *Xenopus laevis* embryos were vegetally injected with 0.25 ng of β-catenin mRNA, 0.5 ng of GR-LEFΔN-βCTA mRNA or 3.5 picomoles of *ndrg1a* MO1 at 4-cell stage, treated with 10 µM Dex at stage 11, and subjected to RT-PCR analysis of *atf3* expression at stage 30. *Ornithine decarboxylase* (*odc*) was used as the RNA loading control. (B, C) *Xenopus laevis* embryos were vegetally injected with 0.3 ng of *atf3* mRNA at 4-cell stage and collected at stages 36 (B) and 42 (C) for whole mount staining with probes indicated on the left side. (B5, 6) Dorsal view. The dorsal structures, such as the neural tube, notochord, and somites were removed after whole mount in situ hybridization. All the rest images in B and C are lateral view with head toward the left. The numbers of embryos showing the illustrated phenotypes are given in the corresponding images. Abbreviation: CE, control embryos.

**Figure 9:**
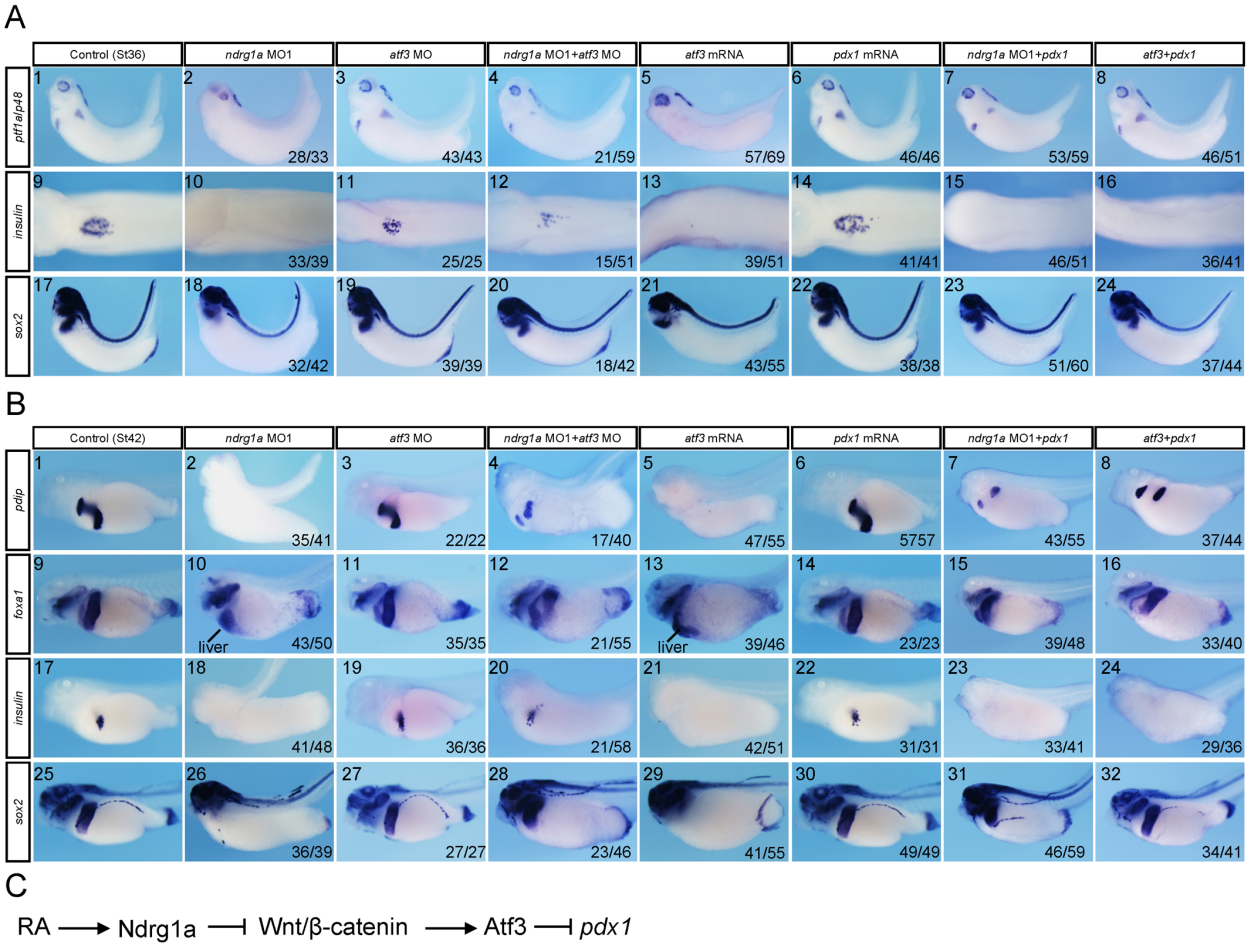
*atf3* MO was able to rescue *ndrg1a* knockdown phenotype. Pdx1 partially rescued *ndrg1a* knockdown or Atf3 overexpression phenotypes. (A, B) *Xenopus laevis* embryos were vegetally injected with the reagents indicated on the top at 4-cell stage and collected at stages 36 (A) and 42 (B) for whole mount staining with probes indicated on the left side. The doses of the reagents injected are as follows: *atf3* MO, 2 picomoles; *pdx1* mRNA, 75 pg; *ndrg1a* MO1, 3.5 picomoles; *atf3* mRNA, 300 pg. (A9–16) Dorsal view. The dorsal structures, such as the neural tube, notochord, and somites were removed after whole mount in situ hybridization. All the rest images in A and B are lateral view with head toward the left. The numbers of embryos showing the illustrated phenotypes are given in the corresponding images. (C) The data obtained suggest an epistasis that RA activated Ndrg1a represses Wnt/β-catenin signaling and consequently releases the inhibitory effect of Wnt/β-catenin, which may be partially mediated by Atf3, on pancreas, oesophagus, stomach, and duodenum formation.

It was shown that ATF3, a Wnt/β-catenin target gene in cancer cell lines [37], can either transcriptionally repress *Pdx1* expression [59] or physically interact with PDX1 to block PDX1 mediated transactivation in a murine β-cell line [60]. During *Xenopus* embryogenesis, *atf3* is hardly detected by whole mount in situ hybridization (Fig. S4). RT-PCR analysis indicated that *atf3* expression was up-regulated upon *ndrg1a* knockdown or β-catenin overexpression in *Xenopus* embryos (Fig. 8A). We found three core Tcf/Lef binding sites (5′-A/T A/T CAAAG-3′) in *Xenopus tropicalis atf3* promoter region, which locate at −1467 bp (TACAAAG), −5591 bp (AACAAAG) and −8889 bp (AACAAAG), respectively. Together, these data suggest that *atf3* is also a Wnt/β-catenin target gene in *Xenopus* embryos. Strikingly, overexpression of Atf3 in *Xenopus* endoderm led to complete loss of *insulin*, *ptf1a*/*p48*, *pdip*, *pdx1*, *foxa1*, and *sox2* expression in the pancreas, oesophagus, stomach, and duodenum, but had no obvious effect on *darmin*, *hhex*, and *nkx2*.*1* expression (Fig. 8B, C), which is just identical to *ndrg1a* knockdown phenotype (Fig. 4A). More importantly, *atf3* MO, which alone caused minor effects on the expression of marker genes analyzed, was able to rescue *insulin*, *ptf1a*/*p48*, *pdip*, *foxa1*, and *sox2* expression inhibited by *ndrg1a* MO (Fig. 9A, B). Furthermore, Pdx1 was able to rescue Atf3 overexpression or Ndrg1a knockdown phenotype with respect to *ptf1a*/*p48*, *pdip*, *foxa1*, and *sox2* expression except for *insulin* (Fig. 9A, B). It is known that Pdx1 is neither necessary nor sufficient for the activation of early *insulin* expression in *Xenopus* embryos [44]. Altogether, our data suggest an epistasis that RA activated Ndrg1a represses Wnt/β-catenin signaling and consequently releases the suppression activity of Wnt/β-catenin signaling activities, which may be partially mediated by Atf3, on pancreas, oesophagus, stomach, and duodenum formation.

## Discussion

*ndrg1a* is directly activated by RA in the archenteron roof endoderm cells of *Xenopus* neurulae. Nuclear β-catenin level is very low in the archenteron roof endoderm cells and it appears that RA activated Ndrg1a represses Wnt/β-catenin signaling in these cells. Knockdown of *ndrg1a* mimics inhibition of RA signaling or activation of Wnt/β-catenin signaling in *Xenopus* embryos with respect to the pancreas, oesophagus, stomach, and duodenum development, which leads to an almost complete loss of the specification of those organ primordia. Blocking Wnt/β-catenin signaling can rescue *ndrg1a* knockdown phenotype. Thus, Ndrg1a coordinates RA and Wnt/β-catenin signaling in the specification of *Xenopus* pancreas, oesophagus, stomach, and duodenum.

### *ndrg1a* might be a Specific Marker Gene for Dorsal Pancreas, Oesophagus, Stomach, and Duodenum Precursors in *Xenopus* Neurulae

Several studies have described microarray based screening of RA responsive genes in *Xenopus* [61] or zebrafish [17], [18], [62] embryos. Using microarray in combination with whole mount in situ hybridization, we were able to identify archenteron roof endoderm specific gene *ndrg1a.* A vital dye staining based fate mapping study has shown that archenteron roof endoderm cells later give rise to dorsal pancreas, oesophagus, stomach, and duodenum [5]. *shirin* appears to be expressed in *Xenopus* archenteron roof endoderm, but meanwhile it is also expressed in mesoderm and ectoderm [63]. *fetuinish* specifically marks the archenteron roof endoderm in *Xenopus* early neurulae, but later it displays expression in liver and intestine with no expression in pancreas, oesophagus, stomach, and duodenum [35]. Only *ndrg1a* shows consistent expression early in the archenteron roof endoderm and later in pancreas, oesophagus, stomach, and duodenum. We speculate that *ndrg1a* can serve as a specific dorsal pancreas, oesophagus, stomach, and duodenum precursor marker gene in *Xenopus*, which remains to be verified by genetic lineage tracing studies.

### Ndrg1a is Indispensable for *Xenopus* Pancreas, Oesophagus, Stomach, and Duodenum Specification

Early endodermal expression of *cyp26a1* and *cdx4* in zebrafish embryos appears to counteract RA signaling from mesoderm to set anterior and posterior boundaries of pancreatic territory respectively [17], [64]. *ndrg1a* is the first gene identified that is directly activated by RA early in *Xenopus* archenteron roof endoderm cells and it mediates RA signaling to positively pattern endoderm cells into pancreas, oesophagus, stomach, and duodenum. Both MO based knockdown of *ndrg1a* and TALEN mediated disruption of *ndrg1a* demonstrate that the Ndrg1a activity in *Xenopus* endoderm patterning is indispensable, which was not observed in *Ndrg1* knockout mice [32], [33], [34]. One explanation for this discrepancy is that the essential role of Ndrg1a in *Xenopus* endodermal patterning is not conserved in mammals. Alternatively, no endodermal organ defects reported in mouse *Ndrg1* gene targeting studies might be due to leaky expression of NDRG1 in *Ndrg1* deficient mice [32], [33] or due to functional redundancy among NDRG family members 1–4 in mice.

A number of studies indicate that overexpression of NDRG1 in cancer cell lines generates conspicuous phenotypes [27]. It is also reported that overexpression of Ndrg1a in *Xenopus* embryos results in a reduced pronephros and disorganized somites [36]. For reasons unknown, we could not get ectopic Ndrg1a in function in *Xenopus* embryos. It should be pointed out that a truncated version of Ndrg1a with the first 48 amino acids in the N-terminal missing was used for both mRNA injection and MO design in the earlier study [36]. We also tried mRNA injection with this short version and the injected embryos were healthy even at high dose (4 ng of mRNA). We could not rescue our *ndrg1a* MO phenotype with this truncated version of Ndrg1a either. Lastly, the strong endodermal expression of *ndrg1a* illustrated in our study and described by Costa et al. [35] was not detected in that study [36].

### Ndrg1a Mediated Crosstalk between RA and Wnt/β-catenin Signaling is Restricted to *Xenopus* Pancreas, Oesophagus, Stomach, and Duodenum Forming Cells

During embryogenesis, cells must constantly integrate multiple signaling pathways to achieve their distinct fate at the right time and place. The integrated role of FGF, RA, and Wnt signaling pathways in specifying lung primordium and controlling lung bud growth defined in mice [65] is conserved in *Xenopus* [66]. In mice, it seems that RA represses Wnt antagonist *Dkk1* expression in the prospective lung endoderm of E8.5–E9.5 embryos, thus allows canonical Wnt signaling mediated pulmonary specification [65]. RA and Wnt pathways are linked by RA downstream target genes *osr1* and *osr2* to maintain pectoral fin development in zebrafish [67]. Together, these cases reveal a circumstance that RA promotes Wnt/β-catenin signaling.

Here, we provide convincing evidence showing for the first time, to our knowledge, that RA activated endodermal expression of Ndrg1a represses Wnt/β-catenin signaling and consequently releases the suppression activity of Wnt/β-catenin signaling on *Xenopus* pancreas, oesophagus, stomach, and duodenum formation, which does not apply to the liver and lung specification. In support of our finding, overexpression of a secreted Wnt antagonist Sfrp5 can substitute for RA in respect to the induction of exocrine pancreas differentiation in VegT injected animal caps in *Xenopus* [24]. The detrimental effects of early ectopic Wnt/β-catenin signaling activation on the liver and lung bud formation seen in this study as well as in an earlier study [22] is not contradictory to the positive role of canonical Wnt signaling in promoting liver and lung development, which was observed when Wnt/β-catenin signaling was activated in *Xenopus* embryos from stages 30 to 42 for liver and from stages 28 to 35 for lung, respectively [22], [66]. The repression effect of RA on *Xenopus* blastula Wnt/β-catenin signaling [26] is unlikely mediated by Ndrg1a, since *ndrg1a* is not maternally provided and its zygotic expression is not activated before neurulation, as revealed by both whole mount in situ hybridization and RT-PCR analyses (Fig. 1A, [36] and data not shown). It remains to be investigated if Ndrg1a can interact with Lrp6 and how Ndrg1a represses Wnt/β-catenin signaling in *Xenopus* embryos.

## Materials and Methods

### Ethics Statement

This study was carried out in strict accordance with the recommendations in the Guide for the Care and Use of Laboratory Animals of the National Institutes of Health. The protocol (2010052) was approved by the Institutional Animal Care and Use Committee (IACUC) of Guangzhou Institutes of Biomedicine and Health, Chinese Academy of Sciences. All surgery was performed under sodium pentobarbital anesthesia, and all efforts were made to minimize suffering.

### Embryo Cultivation and Microarray Analysis

Wild type *Xenopus laevis* or *Xenopus tropicalis* embryos were obtained by in vitro fertilization and staged according to the normal table of *Xenopus* development [68]. *Xenopus laevis* embryos were treated with 0.25 µM BMS453 (a gift from Bristol Myers Squibb) for one hour at stage 10. Corresponding amount DMSO was added to control embryos. Embryos were collected at stages 12, 23, and 34. Total RNA was extracted using TRIZOL reagent (Invitrogen), purified using Qiagen RNAeasy kit, and subjected to microarray analyses using *Xenopus laevis* Genome Arrays (Affymetrix, version 2.0). The array was not repeated. We randomly chose 15 down-regulated genes (Table S1) and validated their expression by RT-PCR. Indeed, all the tested genes showed down-regulation in the BMS453 treated embryos. The raw and normalized data were stored in the ArrayExpress database (accession no. E-MTAB-1419).

### Whole Mount in situ Hybridization and Immunofluorescence

Whole mount in situ hybridization was performed as described [69]. The antisense probes for *darmin*, *for1*, *foxa1*, *insulin*, *hhex*, *pdip*, *pdx1*, and *ptf1a*/*p48* were prepared as described previously [10], [22], [44], [49]. *ndrg1a* (GenBank accession no. NM_001094390), *atf3* (GenBank accession no. NM_001094018), *sox2* (GenBank accession no. NM_213704), and *nkx2*.*1* (GenBank accession no. AF281080) were cloned into pGEM-T Easy vector by RT-PCR. The resultant constructs were cut with SalI and transcribed with T7 RNA polymerase to get their antisense probes.

Immunofluorescence was performed as described [55] with following modifications. Stage 20 embryos were fixed in MEMFA (0.1 M MOPS, pH 7.4; 2 mM EGTA; 1 mM MgSO4 and 4% formaldehyde) for two hours at room temperature, embedded in paraffin, and sectioned sagittally at a thickness of 5 µm. Serial sections were incubated with rabbit anti-β-catenin antibody (1∶250, H-102, Santa Cruz Biotechnology, #sc-7199) and followed by goat anti-rabbit Cy3 (1∶200, Bi Yuntian, #A0516) as a secondary antibody. The nuclei were counterstained with DAPI (1 µg/ml, Roche, #10-236-276-001). Fluorescent images were captured using a Zeiss LSM 710 confocal microscope with 40× objectives.

### MO and mRNA Injection in *Xenopus laevis* Embryos

*ndrg1a* MO1 (5′-ATAGCCGTTTGCCTGTGTAAGAAGA-3′), MO2 (5′-ATACCCTGGTGTCCTGATGCTGCGC-3′), *atf3* MO (5′-AGCATCATTTTCGTGCTGTGTCGGT-3′), and standard control morpholino oligonucleotide (5′-CCTCTTACCTCAGTTACAATTTATA -3′) were purchased from GeneTools, LLC. The open reading frames of *Xenopus laevis ndrg1a*, *atf3* and *pdx1* were obtained by RT-PCR and cloned into the pCS2+ vector. pCS2+HA-GR-ΔNLEF-βCTA [58] and pT7TSHA-GR-ΔNTcf3 [56] were obtained from Zorn laboratory. Capped mRNA was generated with SP6 or T7 mMessage mMechine Kit (Ambion) and purified using Qiagen RNAeasy kit. All the reagents were injected into four blastomeres of the 4-cell stage embryos from the vegetal pole.

### Construction and Application of *Xenopus tropicalis ndrg1* TALENs

A pair of *Xenopus tropicalis ndrg1* TALENs was constructed through Golden Gate TALEN Assembly method [54], [70]. The resultant *ndrg1* TALEN mRNAs were injected into animal pole of fertilized *Xenopus tropicalis* eggs. Five injected embryos were collected at stage 40 for somatic gene targeting analysis. The rest were either collected for marker gene expression analysis, or raised for late phenotyping and establishing of stable knockout lines. Primer 1 (5′-GTGCTGCAAGTTGGAGTGAT-3′) and primer 2 (5′-ACTCTAGGTGGCATGACAGC-3′), bridging the right and left binding sites of *ndrg1* TALENs in wild type *Xenopus tropicalis* genomic DNA, were used to amplify the targeting region of *ndrg1*. The obtained PCR fragments were subcloned to pGEM-T Easy vector and single colonies were picked for sequencing.

### Chemical Treatment

RA (all-trans-RA, Sigma) and cycloheximide (CHX, Sigma) were first prepared as 10 mM and 10 mg/ml stock solutions in 100% ethanol and then diluted into desired concentrations with 0.1×MBS (at least 1∶1000 dilution). Carrier controls were performed at the highest solvent concentration that the experimental embryos received in each set. For the activation of GR fusion proteins, dexamethasone (Dex, Sigma) was prepared as 5 mM stock solution in 100% ethanol and applied to the control and mRNA-injected embryos at stage 11 in a final concentration of 10 µM in 0.1× MBS.

### RT-PCR

RT-PCR was carried out as previously described [10]. The following primers and cycle numbers were used: *atf3* (forward 5′-TTTAGATTCGGTGGTGGTGTCC-3′ and reverse 5′-ATCTGCTGGATGAAGAGGTTGC-3′, 28cycles), and *ornithine decarboxylase* (forward 5′-TGAATTGATGAAAGTGGCAAGG-3′ and reverse 5′-CAGGGCTGGGTTTATCACAGAT-3′, 23cycles).

## Supporting Information

Figure S1 **The down-regulated genes upon BMS453 treatment appear dynamic during early development.** A Venn diagram indicates that only a few genes showing overlapping at different stages of development. The 9 genes selected for further whole mount in situ analysis are listed in the diagram.
(TIF)

Figure S2 **Injection of** ***ndrg1a*** **mRNA into** ***Xenopus*** **embryos generated no phenotype.** *Xenopus laevis* embryos were vegetally injected with 4 ng of *ndrg1a* mRNA at 4-cell stage and collected at stages 36 (A) and 42 (B) for whole mount staining with probes indicated on the left side. (A5, 6) Dorsal view. The dorsal structures, such as the neural tube, notochord, and somites were removed after whole mount in situ hybridization. All the rest images in A and B are lateral view with head toward the left. The numbers of embryos manipulated are given in the individual images.
(TIF)

Figure S3 **Ndrg1a-GFP fusion protein was properly synthesized in** ***Xenopus*** **embryos.** 1 ng of *ndrg1a*-*GFP* mRNA was injected into the vegetal part of all four blastomeres of 4-cell stage *Xenopus laevis* embryos. (A) Live GFP signals were observed under an Olympus SZX16 fluorescence microscope when the injected embryos developed to stage 35. (B) Western blot analysis with an anti-GFP antibody confirmed that fusion protein was properly generated in stage 35 embryos. For Western blot analysis, in addition to the control uninjected embryos, we also injected GFP mRNA as a control. CE, control embryos.
(TIF)

Figure S4 ***atf3*** **expression is nearly undetectable in developing** ***Xenopus*** **embryos by whole mount in situ hybridization.** All images are lateral view. (C–G) Head toward the left.
(TIF)

Table S1 **List of genes down regulated more than 2-fold in BMS453 treated** ***Xenopus laevis*** **embryos collected at stages 12, 23, and 34 based on one Affymetrix Genome Array.** The overlapping ones among three stages analyzed are crossed in additional columns. The 9 genes that were chosen for further in situ hybridization analysis are highlighted in bold.
(XLS)
