# Supplementary figures and images for: Retinoic Acid-Activated Ndrg1a Represses Wnt/β-catenin Signaling to Allow Xenopus Pancreas, Oesophagus, Stomach, and Duodenum Specification
Source: PLoS One. 2013 May 31;8(5):e65058. doi: 10.1371/journal.pone.0065058 (PMC3669096; doi:10.1371/journal.pone.0065058)

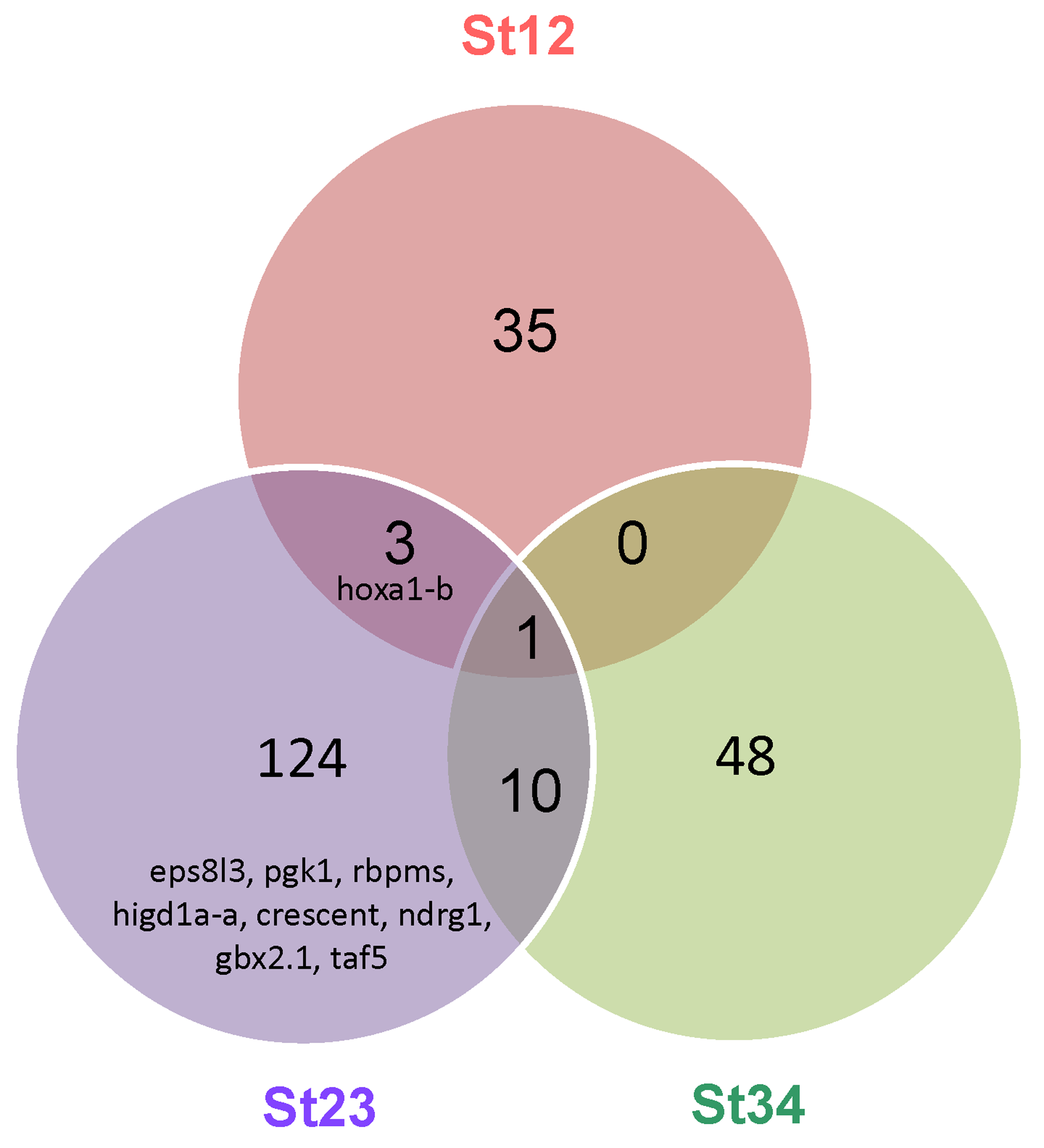

Supplement: Figure S1 — The down-regulated genes upon BMS453 treatment appear dynamic during early development. A Venn diagram indicates that only a few genes showing overlapping at different stages of development. The 9 genes selected for further whole mount in situ analysis are listed in the diagram. (TIF) [file pone.0065058.s001.tif]

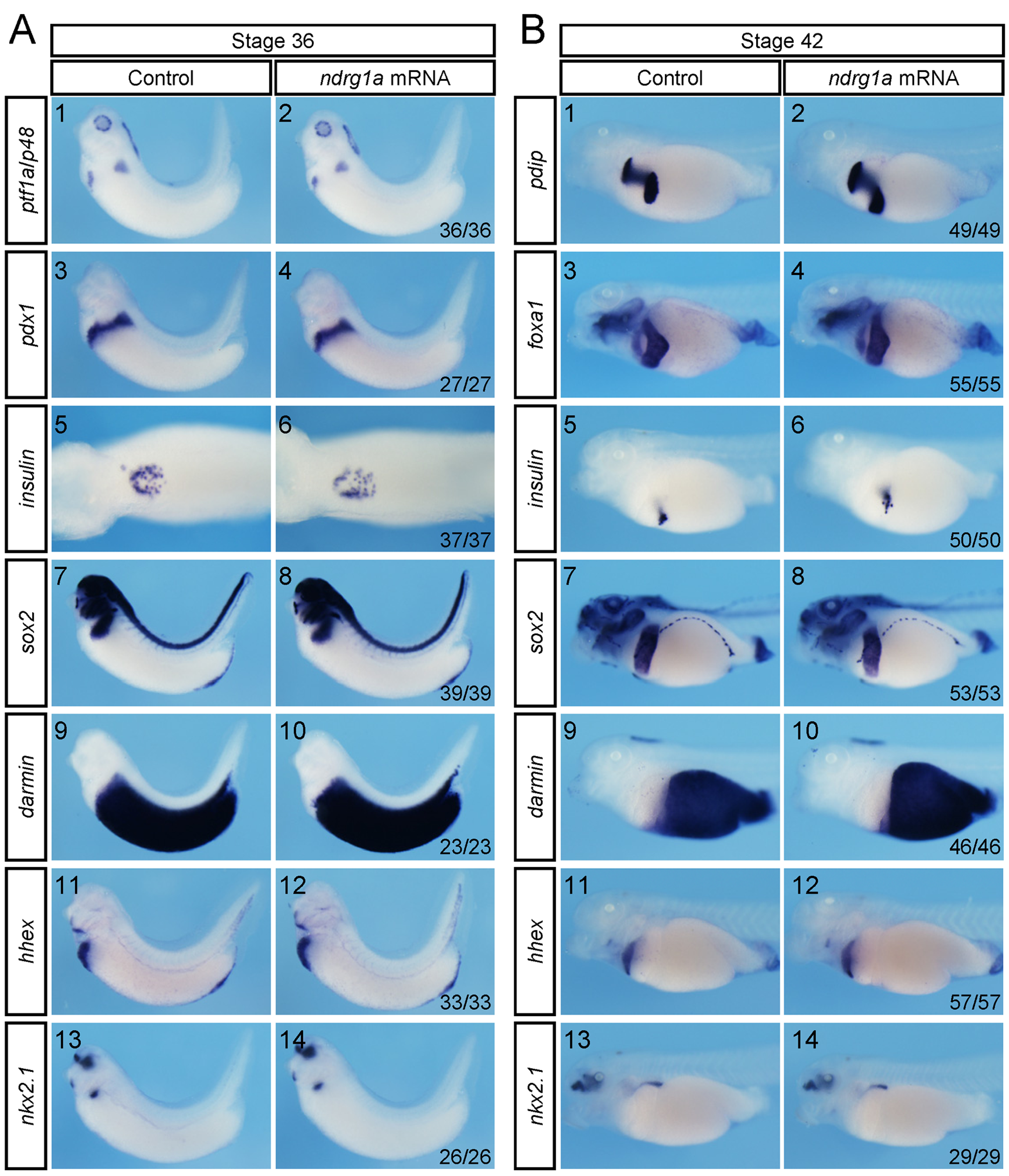

Supplement: Figure S2 — Injection of ndrg1a mRNA into Xenopus embryos generated no phenotype. Xenopus laevis embryos were vegetally injected with 4 ng of ndrg1a mRNA at 4-cell stage and collected at stages 36 (A) and 42 (B) for whole mount staining with probes indicated on the left side. (A5, 6) Dorsal view. The dorsal structures, such as the neural tube, notochord, and somites were removed after whole mount in situ hybridization. All the rest images in A and B are lateral view with head toward the left. The numbers of embryos manipulated are given in the individual images. (TIF) [file pone.0065058.s002.tif]

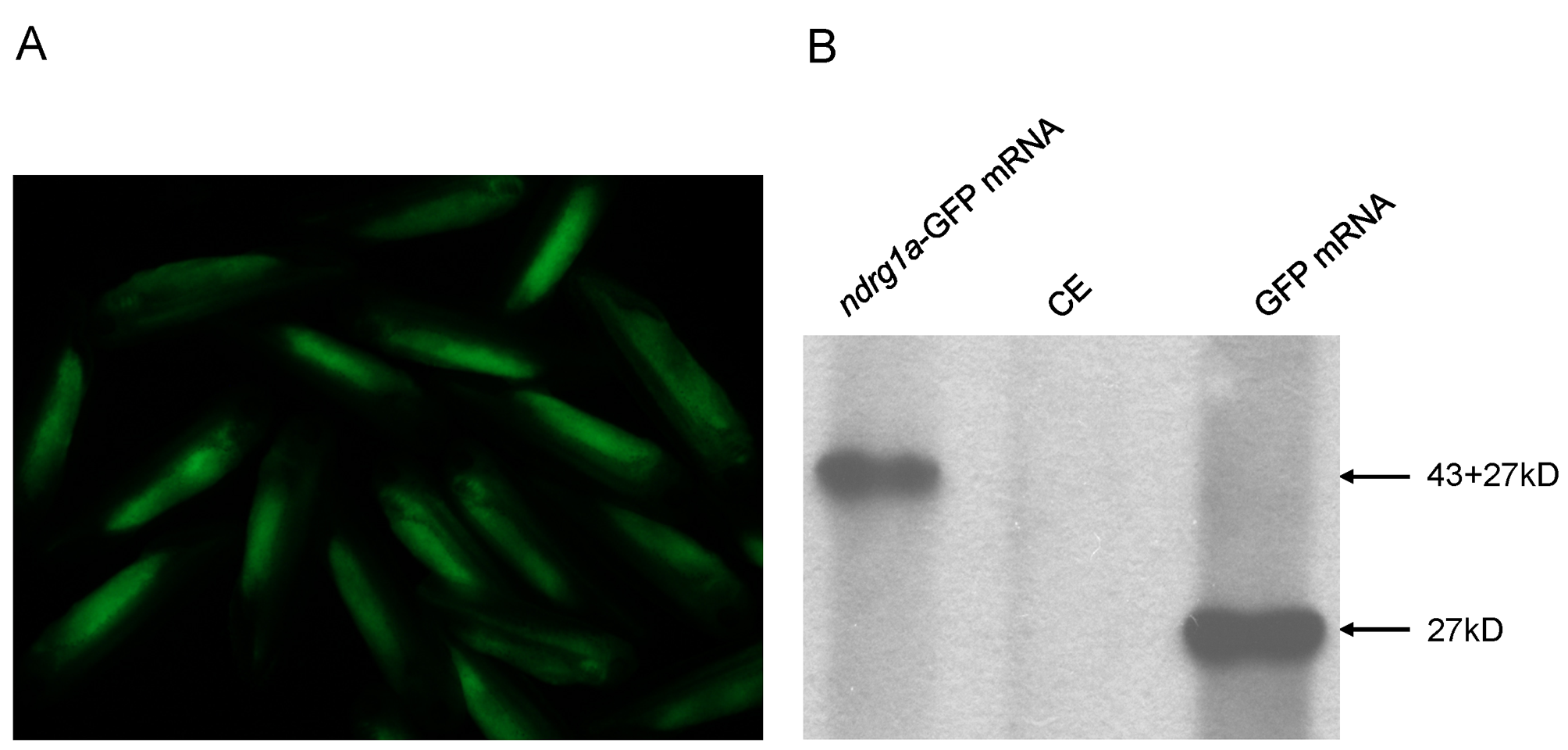

Supplement: Figure S3 — Ndrg1a-GFP fusion protein was properly synthesized in Xenopus embryos. 1 ng of ndrg1a-GFP mRNA was injected into the vegetal part of all four blastomeres of 4-cell stage Xenopus laevis embryos. (A) Live GFP signals were observed under an Olympus SZX16 fluorescence microscope when the injected embryos developed to stage 35. (B) Western blot analysis with an anti-GFP antibody confirmed that fusion protein was properly generated in stage 35 embryos. For Western blot analysis, in addition to the control uninjected embryos, we also injected GFP mRNA as a control. CE, control embryos. (TIF) [file pone.0065058.s003.tif]

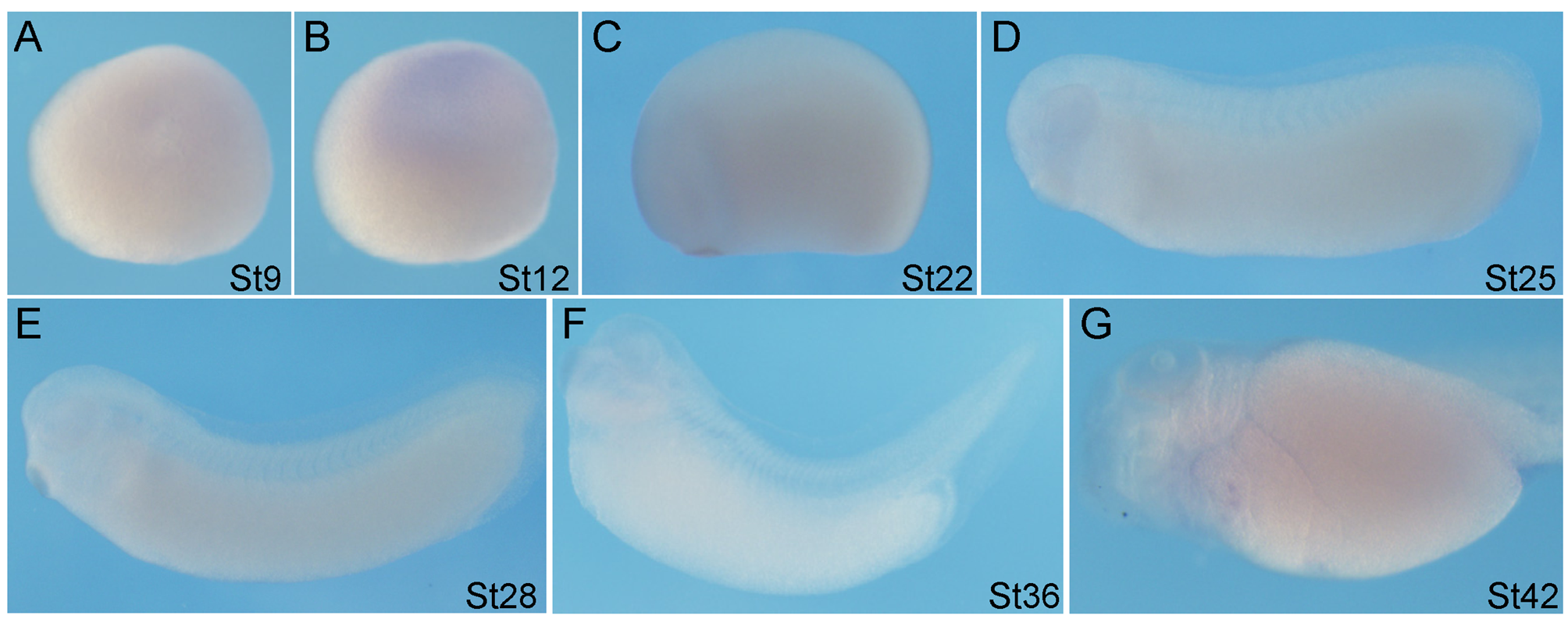

Supplement: Figure S4 — atf3 expression is nearly undetectable in developing Xenopus embryos by whole mount in situ hybridization. All images are lateral view. (C–G) Head toward the left. (TIF) [file pone.0065058.s004.tif]
